# A *De Novo FOXP1* Truncating Mutation in a Patient Originally Diagnosed as C Syndrome

**DOI:** 10.1038/s41598-017-19109-9

**Published:** 2018-01-12

**Authors:** Roser Urreizti, Sarah Damanti, Carla Esteve, Héctor Franco-Valls, Laura Castilla-Vallmanya, Raul Tonda, Bru Cormand, Lluïsa Vilageliu, John M. Opitz, Giovanni Neri, Daniel Grinberg, Susana Balcells

**Affiliations:** 1 Department of Genetics, Microbiology and Statistics, Faculty of Biology, University of Barcelona, IBUB, IRSJD, CIBERER, Barcelona, Spain; 2 Geriatric Unit, Fondazione Ca’Granda, IRCCS Ospedale Maggiore Policlinico, Milan, Italy; 3 Nutritional Sciences, University of Milan, Milan, Italy; 4 CNAG-CRG, Centre for Genomic Regulation (CRG), Barcelona Institute of Science and Technology (BIST), Barcelona, Spain; 5 Universitat Pompeu Fabra (UPF), Barcelona, Spain; 6 Pediatrics Medical Genetics, University of Utah School of Medicine, Salt Lake City, Utah USA; 7 Istituto di Medicina Genomica, Università Cattolica Sacro Cuore, Policlinico A. Gemelli, Rome, Italy

## Abstract

*De novo FOXP1* mutations have been associated with intellectual disability (ID), motor delay, autistic features and a wide spectrum of speech difficulties. C syndrome (Opitz C trigonocephaly syndrome) is a rare and genetically heterogeneous condition, characterized by trigonocephaly, craniofacial anomalies and ID. Several different chromosome deletions and and point mutations in distinct genes have been associated with the disease in patients originally diagnosed as Opitz C. By whole exome sequencing we identified a *de novo* splicing mutation in *FOXP1* in a patient, initially diagnosed as C syndrome, who suffers from syndromic intellectual disability with trigonocephaly. The mutation (c.1428 + 1 G > A) promotes the skipping of exon 16, a frameshift and a premature STOP codon (p.Ala450GLyfs*13), as assessed by a minigene strategy. The patient reported here shares speech difficulties, intellectual disability and autistic features with other FOXP1 syndrome patients, and thus the diagnosis for this patient should be changed. Finally, since trigonocephaly has not been previously reported in FOXP1 syndrome, it remains to be proved whether it may be associated with the FOXP1 mutation.

## Introduction

Severe neurodevelopmental disorders (NDD) affect more than 3% of children and are due to a genetic defect in more than 80% of the cases^1^. Until recently, the main diagnostic tools include a great variety of molecular tests (karyotype, array CGH and Sanger sequencing of putative candidate genes) together with multiple clinical evaluations by highly specialized physicians and a variety of complementary tests, some of them invasive, such as neuroimaging, metabolic evaluation or cerebrospinal fluid examination^1^. After this “diagnostic odyssey” more than a half of the patients are still miss- or undiagnosed^2^ and this situation is worsened in patients suffering from rare diseases clinically and genetically heterogeneous or with unclear or atypical presentations^2,3^. Next-generation sequencing (NGS) has revolutionized the field of clinical genetics by highly improving the diagnostic yield of rare diseases, and facilitating the identification novel causative genes^3^, achieving a molecular diagnosis in 25–68% of the cases^4^.

**Figure 1:**
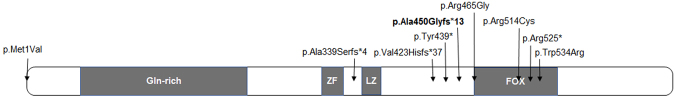
FOXP1 domain organization (for UniProt Q9H334). Point mutations associated with intellectual disability (ID) with language delay, with or without autistic features are shown. In bold, the mutation found in the patient described here.

*FOXP1* is one of the genes recently found mutated in a Mendelian developmental disorder. Heterozygous sequence variants have been linked to intellectual disability (ID) with language delay, with or without autistic features (MIM #613670). The FOXP1 (forkhead-box protein P1; MIM 605515) protein is a member of the forkhead-box family of transcription factors characterized by a highly conserved forkhead DNA-binding domain (FOX) and with crucial roles in embryonic development^5,6^. The FOXP1 protein, as other members of the FOXP family, includes 4 main functional domains: a N-terminal glutamine (Gln) rich region, zinc finger and leucine zipper domains and a C-terminal FOX domain (Fig. 1).

*FOXP1* is important in neural development^7^, monocyte differentiation and macrophage function^5^. It has also been described as an oncogene in hepatocellular carcinoma, pancreatic cancer, and various types of B-cell non-Hodkgin lymphomas^8^, being yet another example of the tight connection between cancer-related genes and developmental defects^9^. Most FOX proteins bind to their target DNA sequences as monomers, except members of the FOXP subfamily, which include FOXP1–4. FOXP1 is able to form both homo and heterodimers (with its paralogous FOXP2) via the leucine zipper domain^10,11^. In mouse, *Foxp1* and *Foxp2* have been shown to be co-expressed in several brain regions^12^ as well as in perichondrial skeletal progenitors and proliferating chondrocytes during endochondral ossification and they act as coordinators of osteogenesis and chondrocyte hypertrophy in the developing long bones^13^. In humans, FOXP2 is involved in a rare form of speech and language disorder with developmental verbal dyspraxia (childhood apraxia of speech or CAS, MIM #602081).

Here we present an adult patient with developmental delay, trigonocephaly, speech impairment and ID who was diagnosed as Opitz C syndrome early in life, in whom we have identified by WES a novel splicing mutation in the *FOXP1* gene and provide a detailed clinical description, in comparison to so far reported patients identified carrying deleterious *FOXP1* mutations.

## Results and Discussion

The patient described here (Patient 2 in Urreizti *et al*.^14^) is a 23-year-old man, second child of non-consanguineous Italian parents. Pregnancy was complicated by threatened abortion. The delivery was vaginal at 40 weeks. Birth weight was 3100 g (25th centile) and length 50 cm (25th centile). Apgar scores were 9 and 10, at 1 and 5 minutes, respectively. Patient’s karyotype was normal (46,XY), and so were array CGH (at an average resolution of 75Kb) and *FMR1* sequencing.

**Figure 2:**
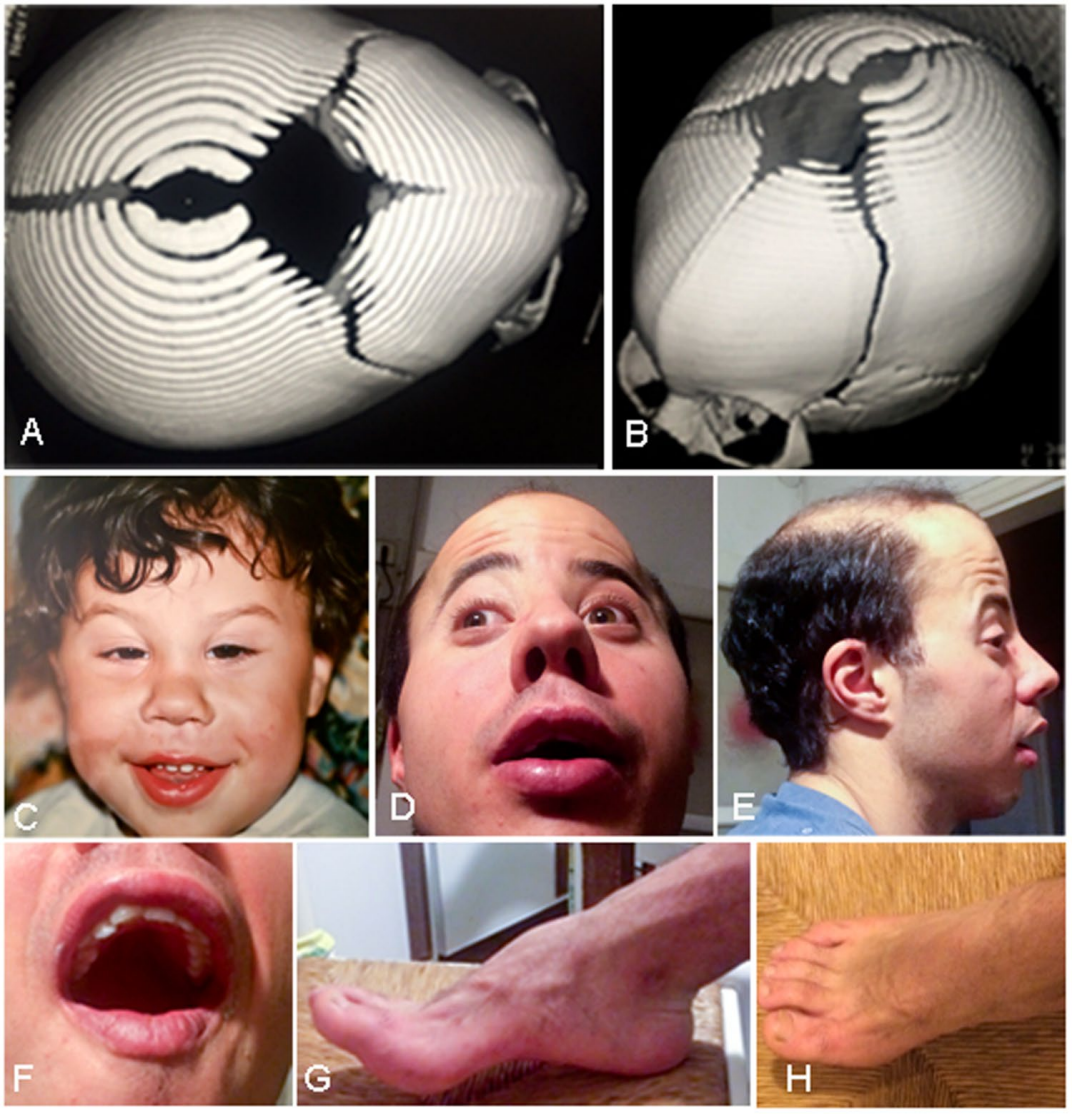
Main features of the patient. (**A**–**B**) Neonatal CT scan. Trigonocephaly is clearly appreciated. For a control CT scan, see Khanna *et al*.^32^ (**C**–**E**) Facial dysmorphisms at 2 and 23 years of age. Hypertelorism, convergent strabismus, down slanted palpebral fissures, epicanthus, prominent nasal root and ante-verted nostrils and (**F**) arched palate and hypertrophy of the alveolar processes are appreciated. (**G**–**H**) Foot phenotype with metatarsal flat-footedness and clinodactyly. All images are shared by the family with explicit permission to publish.

He presented with mild macrocephaly (44.5 cm at 4 months and 55 cm at 8 years, which correspond to +1.74 and +2.0 SD, respectively), peculiar facial appearance (Fig. 2) and premature closure of the metopic suture with trigonocephaly (Fig. 2A,B), marked hypertelorism, convergent strabismus, downslanted palpebral fissures, epicanthus, blepharophimosis (since the first months of life), prominent nasal root and anteverted nostrils (Fig. 2C,D), hyperconvolute helix, low-set and posteriorly angulated ears (Fig. 2E), hypoplasia of the malar region, micrognathia, highly arched palate and hypertrophy of the alveolar ridges (Fig. 2F). The patient also had large hands and long fingers, horseshoe kidney, bilateral cryptorchidism, slight deformity of the forefoot with flat-footedness in metatarsal level and clinodactyly of the fifth toes (Fig. 2G,H). He also presented with astigmatism and hypermetropia and had a tendency to form cutaneous keloids. The patient had marked oral and lingual dyspraxia and a reduced facial mimicry. As a child, he manifested enuresis and hyperphagia. He underwent: skull remodeling with bilateral frontal advancement and re-alignment of the orbital bar, correction of strabismus and cryptorchidism and adeno-tonsillectomy for snoring. He is relatively short (158 cm whereas his father’s height is 185 cm).

He was able to sit at 11 months, did not crawl and maintained orthostatism at 14 months, with instability and frequent falls. Thereafter, there was an improvement in static and dynamic balance, albeit with an underlying state of hypertonicity, postural oddities and serious coordination deficits of fine and large movements. He was able to walk autonomously at 3 years, but up to 5 years he was unable to protect himself from falls. From 9 to 12 years he was treated with botulinum intra-muscular injections, with only partial and transient benefit. While growing, the hypertonic state gradually resolved, but mobility remained clumsy, with toe-walking and an altered dynamic equilibrium (use of the trunk movement to maintain balance).

The patient displayed a marked developmental delay in all intellectual milestones. First words were at 2 years and complete language was reached at 5 years. During childhood, he had a tendency to repetition and echolalia; over time this improved, but the patient had repetitive speech around the same questions. Expressive language was affected to a greater degree than receptive language. Furthermore, a substantial disorder of attention and cognitive rigidity prevented adequate learning at school. He manifested features of autism spectrum disorders (ASD), such as aggression, hyperactivity, repetitive manipulation of the objects and tendency to explore the environment in a purposeless way. He rejected proposals that did not immediately satisfy his own needs, had reduced social interaction with a childlike and self-interested attitude and a tendency to search for attention and reassurance. ID was of severe degree (IQ 33), with concrete thinking and partial temporal orientation.

The patient was initially diagnosed as C Syndrome (CS, Opitz C trigonocephaly, MIM #211750), a rare condition whose etiology is still poorly understood and whose diagnosis is still only clinical. CS was initially proposed because of trigonocephaly, epicanthus, strabismus, micrognathia, ear anomalies, renal anomalies and severe ID. On the other hand, some of the patient’s characteristics did not fit into the C syndrome^15^: hypertelorism (whereas in CS hypotelorism is typical), macrocephaly (instead of microcephaly), downslanted palpebral fissures (instead of upslanted) and hypertone (instead of hypotone). After a clinical evaluation at 20 years of age, the patient’s diagnosis was changed to “unknown”.

We performed whole-exome sequencing on the patient’s genomic DNA after obtaining written informed consent and the approval of the protocol by the Ethics Committee of the Universitat de Barcelona. We found a novel *de novo* truncating mutation in the *FOXP1* gene (c.1428 + 1 G > A) in heterozygosis. In addition, we found several variants of unknown significance (VUS) in other genes (reported and commented in Table S1). All these variants were inherited from either one of his healthy parents and none seems related to the major clinical outcomes of the patient. Of note, the patient is homozygous for the missense mutation p.Ala632Thr (rs72468667) at the *ASXL1* gene. Truncating mutations in this gene are the main cause of Bohring-Opitz syndrome (BOS, MIM #605039), characterized by intellectual disability and trigonocephaly (present in the patient reported here), together with intrauterine growth retardation, feeding difficulties and failure to thrive, and a typical posture (BOS posture), none of which are present in our patient. So far, no missense mutations have been found associated with BOS. In addition, mutation p.Ala632Thr has been found in the general population in 415 out of 277,188 individuals, including one homozygous individual (data from GenomeAD). Whether this missense mutation might contribute to the trigonocephaly of the patient remains an open question.

The *FOXP1* mutation found in the patient consists on a c.1428 + 1 G > A transition at the donor splice site of intron 16. By means of a minigene strategy, we demonstrated that this change leads to the skipping of exon 16 and to a new reading frame starting at alanine 450 and a premature STOP codon after 13 residues (p.Ala450Glyfs*13). This truncated protein would lack 214 residues at the C-terminus, including the FOX domain (Fig. 1). Functional studies performed on similar truncating mutations (also lacking the terminal FOX domain of the protein) showed that they were retained in the cytoplasm^16,17^ and were unable to repress transcription, as demonstrated by luciferase reported assays. Also, as the majority of the truncating mutations retain the leucine zipper domain, necessary for homo- and heterodimerization (see Fig. 1), they bind the wt FOXP1 and FOXP2 proteins, sequestering them in the cytoplasm. This interaction could lead to a dominant negative effect. On the other hand, Sollis *et al*.^17^ found that two truncating mutations (p.Ala339Serfs*4 and p.Arg525*) and the missense mutation p.Trp534Arg were unable to interact with wt FOXP1 or FOXP2 and the nuclear localization of the wt FOXP1 and FOXP2 proteins was unperturbed, ruling out dominant negative effects for these mutations. Additionally, it has been described that some of the alleles bearing truncating mutations are degraded by the nonsense-mediated mRNA decay (NMD)^16,18^. Finally, several cases have been described with deletions or balanced translocations affecting just the *FOXP1* gene^19–23^. A mechanism of loss of function and haploinsufficiency seems to be the common feature of all mutations associated with *FOXP1* disorders, including the one described in our patient.

Table 1 summarizes the main clinical features of FOXP1 patients with published clinical data. It includes point mutations (Fig. 1) or small deletions affecting only the *FOXP1* gene. In agreement with these patients, the patient reported here presented with speech and language impairment (with expressive language more severely affected than receptive language) as well as ID and delay in all motor milestones. He also presented with mild macrocephaly and hypertelorism, a trait commonly observed in these patients. Autism was diagnosed in 3 of 8 patients. However, autistic features and/or behavioral problems were observed in all but one of the reported patients, including the present one who presented with impulsivity, stereotypic behaviors and reduced social interest, among other autistic traits. This is in agreement with the reduced exploratory attitudes and increased evasion of social contact, not due to anxiety, observed in mice with a conditional deletion of *Foxp1* in brain^24^. Other features shared by the present patient and some of the other FOXP1 cases include relatively short stature, strabismus, high-arched palate and enuresis^16,17,25,26^.

**Table 1:** Clinical revision of FOXP1 patients.

**Symptoms**	**Palumbo 2013**	**Le Fevre 2013**	**Srivastava 2014**	**Song 2015**	**Lozano 2015**	**Blanco-Sánchez 2015**	**Sollis 2016**	**Current Patient**	**Total**
De novo mutation	+	9/9	+	+	+	+	3/3	+	18/18
Low birthweight	−	0/2	nd	+	nd	nd	nd	+	2/5
FTT or small for age	−	1/5	nd	−	nd	nd	nd	+	2/8
Obesity	−	2/5	nd	−	nd	nd	nd	−	2/8
Prominent forehead	+	4/7	nd	+	−	+	2/3	+^(1)^	10/15
Macrocephaly	−	nd	+	nd	+	−	1/3	mild	4/8
Hypertelorism	−	nd	nd	nd	−	nd	2/3	+	3/6
Down slanted palpebral fissures	+	3/7	nd	−	+	+	1/3	+	8/15
Short nose with broad tip	nd	4/7	nd	+	−	+	2/3	+	9/14
Frontal hair upsweep	nd	2/7	nd	+	−	+	nd	−	4/11
Prominent digit pads	nd	2/7	nd	−	nd	nd	nd	−	2/9
Single palmar creases	nd	1/7	nd	−	nd	nd	nd	−	1/9
Clinodactyly	nd	1/7	nd	−	nd	nd	nd	+	2/9
Congenital anomalies^(2)^	nd	3/8	nd	+	nd	nd	nd	+	5/10
Global delay	+	9/9	nd	+	+	+	3/3	+	17/17
Regression	nd	1/2	nd	-	nd	nd	nd	−	1/4
Intellectual delay	+	8/8	+	+	+	+	3/3	+	17/17
Motor delay	+	8/8	nd	+	+	+	3/3	+	16/16
Speech and language delay	+	9/9	nd	+	+	+	3/3	+	17/17
Expressive language more severely affected than receptive language	nd	7/7	nd	+	+	+	nd	+	11/11
Articulation problems	+	5/5	nd	−	+	nd	3/3	+	11/12
Poor grammar	nd	4/4	nd	−	+	nd	nd	+	6/7
ASD/PDD-NOS	+	3/4	nd	−	+	nd	3/3	+	9/11
Autism	+	2/4	nd	−	+	nd	0/3	−	4/11
Behavioral problems	+	4/5	nd	−	+	nd	3/3	+	10/12
ADHD and/or sensory processing disorders	+	nd	nd	−	+	nd	2/3	+	5/7
Hypertonia	nd	1/2^(3)^	nd	−	−	nd	0/3	+	2/8
Hypotonia	+	1/2^(3)^	+	nd	+	nd	3/3	−	7/9
Reflexes	nd	1/2	nd	−	nd	nd	nd	+	2/4
Seizures	nd	2/6	nd	−	nd	nd	nd	−	2/8

FTT: Failure to Thrive; ASD: Autistic Spectrum Disorders; PDD-NOS: Pervasive Developmental Disorder Not Otherwise Specified; ADHD: Attention Deficit Hyperactivity Disorder; nd = no data. ^(1)^The patient presented with trigonocephaly. ^(2)^Including: contractures, spina bifida, Chiari 1 malformation, jejunal and ileal atresia, bilateral inguinal hernia^25^, bilateral cryptorchidism, horseshoe kidney (current patient) and hyperextension of the joins^26^. ^(3)^One patient in Carr *et al*.^21^ presented with decreased axial tone and increased peripheral tone.

The patient presented here developed blepharophimosis at an early age, same as in the patient described by Pariani *et al*.^27^, who bears a deletion of the 3p14.1p13 region including the *FOXP1, EIF4E3, PROK2* and *GPR27* genes. Our patient also shares epicanthal folds and hypermetropia with the one in Pariani *et al*., but did not display ptosis of the eyelids. In both patients, hypertone was more severe distally and was not associated with muscular spasm or distal contractions of the fingers. However, in our case hypertone was not equally distributed at 4 limbs but was predominant at the legs, and there were neither distal contractures (present in Pariani *et al*.’s^27^ patient) nor neuropathy.

To our knowledge, this is the first case of *FOXP1* mutation associated with trigonocephaly, although other craniofacial anomalies such as prominent forehead are common in FOXP1 patients. Interestingly, FOXP1 negatively regulates the expression of *Runx2*^13^, a master transcription factor essential for bone development, whose haploinsufficiency produces cleidocranial dysplasia, which includes as main features patent sutures and fontanelles. On the other hand, trigonocephaly is a relatively prevalent condition in the general population, and it might be due to an independent concomitant cause in this patient.

In conclusion, we describe a patient presenting with mental retardation, speech and language delay and minor craniofacial dysmorphic features, in whom the genetic cause of the disease, a *de novo* truncating mutation in *FOXP1*, could be identified, putting an end to 23 years of diagnostic uncertainty. To our knowledge, it is the first case of FOXP1 syndrome presenting with true trigonocephaly due to metopic synostosis, expanding the clinical spectrum of this syndrome.

## Material and Methods

### Biological samples

Genomic DNA from the patient and his parents was obtained from Istituto di Medicina Genomica, Università Cattolica Sacro Cuore, Rome. The signed informed consent was obtained from the patient’s mother, including explicit permission to share clinical and identifying information, including in on-line open-access journals. All protocols were approved by the Ethics Committee of the Universitat de Barcelona and all methods were performed in accordance with the relevant guidelines and regulations.

### Whole exome sequencing and molecular analyses

Whole exome sequencing of the proband was performed in the National Center of Genomic Analysis (CNAG; Barcelona, Spain), using the Illumina HiSeq-2000 platform. Exome capture was performed with Nimblegen SeqCap 64 Mb v3 (Roche; Mannheim; Germany). The samples where sequenced at a coverage of 50X. The data were analyzed as described elsewhere^28^. The results were then filtered under de novo dominance and recessive hypotheses. Variants with a MAF above 0.001 (under the dominant) and above 0.01 (for recessive) in the common population (according to ExAC and 1000 genomes) were excluded. Variants in genes included in selected databases [The Development Disorder Genotype - Phenotype Database (DDG2P)]^29,30^ and covered by at least 10 reads were prioritized for validation (it should be noted that those who carried out the original DECIPHER analysis and collection of the data bear no responsibility for the further analysis or interpretation of it). In parallel, variant effects were classified as high, moderate or low according to SnpEff ^31^ and mutations with a high putative effect and at least 10 reads were also prioritized for validation by Sanger sequencing.

On average, the mean coverage for the patient was of 69 reads, and 93% of the target region was covered by at least 10 reads (C10). A total of 19 variants were selected for validation by Sanger sequencing. Primer sequences and PCR conditions are available on request. PCR reactions, purification and sequencing were performed as described previously^14^.

### Minigene transcript analysis

In order to study the putative effect of the *FOXP1* mutation on splicing, a minigene was constructed, including the genomic region (ENST00000475937) that encompasses exons 15, 16 and 17 (1800pb). This region was PCR-amplified from the patient’s DNA using primers: FOXP1-MG-F (5′-CTCGAGTCCCAACTGGTGTCACCTAA-3′) and FOXP1-MG-R (5′-GGATCCGAGCATTTCAACCACAATGG-3′). These primers include restriction sites for *Xho*I and *BamH*I, respectively (underlined). The wild-type and mutant PCR fragments were cloned into the pSPL3 vector (Addgene). Splicing was assessed 24 h after transfection in HeLa cells. RNA was extracted using the High Pure RNA isolation kit (Roche) and retro-transcribed using the High-Capacity cDNA Reverse Transcription kit (Thermo Fisher Scientific). Primers SD6 and SA2, specific for the pSPL3 vector, were used to amplify the spliced region. The PCR fragments were separated by agarose gel electrophoresis, purified and Sanger sequenced.

## Electronic supplementary material

Supplementary material
