# Supplementary material for: A De Novo FOXP1 Truncating Mutation in a Patient Originally Diagnosed as C Syndrome
Source: Sci Rep. 2018 Jan 12;8:694. doi: 10.1038/s41598-017-19109-9 (PMC5766530; doi:10.1038/s41598-017-19109-9)
Supplement: Supplementary file 1 — Supplementary material [file 41598_2017_19109_MOESM1_ESM.pdf]

A *DE NOVO FOXP1* TRUNCATING MUTATION IN A PATIENT ORIGINALLY DIAGNOSED AS C SYNDROME

Roser Urreizti, Sarah Damanti, Carla Esteve, Héctor Franco-Valls, Laura Castilla-Vallmanya, Raul Tonda, Bru Cormand, Llüisa Vilageliu, John M. Opitz, Giovanni Neri, Daniel Grinberg, Susana Balcells

Supplementary Material.

Supplementary Table S1:

GENE	Change	cDNA	Protein	Inheritance	Disease[1]	MIM number	PT[2]	Provean/SIFT/ PolyPhen2	ExAC MAF (homoz.)	Constrained[3]	Comments
SLC17A5	C>T	c.567G>A	p.Trp189*	Patient htz; father htz	Salla disease<br>Sialic acid storage disorder, infantile	604369<br>269920	AR<br>AR	NA/NA/NA	-	no	Only one putatively pathogenic allele detected in the patient, inherited from an unaffected parent.
POLH	.>AA	c.117insAA	p.Ser40Asnfs*8	Patient htz; mother htz	Xeroderma pigmentosum	278750	AR	NA/NA/NA	-	no	Only one putatively pathogenic allele detected in the patient, inherited from an unaffected parent.
TRPC5	G/T	c.279C>A	p.Ser93Arg	Patient hemiz; mother htz	none			N/T/B	0.00001 (0)	yes (missense and LoF)	rs779715044. Only one putatively pathogenic allele detected in the patient, inherited from an unaffected parent.
MAP4K2	G>A	c.439C>T	p.Gln147*	Patient htz; father htz	none			NA/NA/NA	0.000008 (0)	no	Only one putatively pathogenic allele detected in the patient, inherited from an unaffected parent.
DMD	C>G	c.2971G>C	p.Glu991Asn	Patient hemiz; mother htz	Becker muscular dystrophy<br>Cardiomyopathy, dilated, 3B<br>Duchenne muscular dystrophy	300376<br>302045<br>310200	XLR<br>XL<br>XLR	N/T/D	0.0015 (60)	yes (LoF)	rs72468667: described in hemizygosity in 60 healthy individuals. Inherited from an unaffected parent.
MAGEL2	C>T	c.1894G>A	p.Ala632Thr	Patient htz; mother htz	Schaaf-Yang syndrome (SHFYNG)	615547	AD	NA/NA/NA	0.00003 (0)	Yes (LoF)	*MAGEL2* gene is maternally silenced. The mutation is in the inactive copy of the gene. All mutations previously associated with SHFYNG are truncating.
ASXL1	A>G	c.2957A>G	p.Asn986Ser	Patient hom; both parents are htz	Bohring-Opitz Syndrome (BOS)	605039	AD	N/T/B	0.0012 (0)	no	rs145132837: present in 153 healthy individuals, no homozygous individuals reported. All mutations previously associated with BOS are *de novo* truncating mutations.

HTZ: Heterozygous; Hom: Homozygous; Hemiz: hemizygous, AR: Autosomal Recessive; XLR: X-linked Recessive; XL: X-linked; AD: Autosomal Dominant; NA: Not Available; N: Neutral; T: Tolerated; B: Benign; D: Damaging; LoF: Loss of Function

[1] disease/s associated with mutations in this gene

[2] PT: pattern of inheritance of the associated disease.

[3] Genes "constrained" by evolution according to ExAC database. These genes presented less mutations than expected by chance, suggesting an important function for the gene.

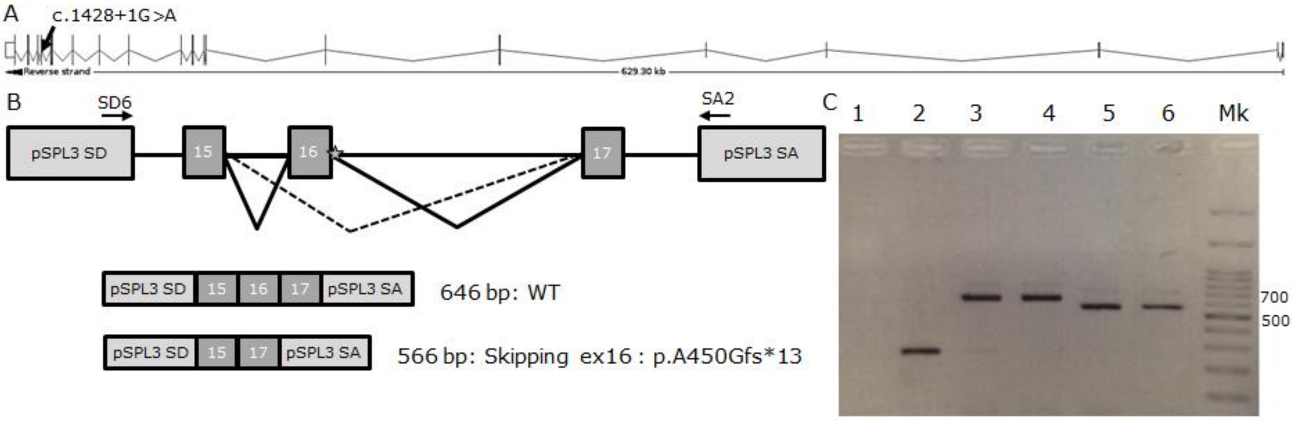

Supplementary Figure S1. (A) *FOXP1* gene representation (ENST00000318789.4). The black arrow marks the position of the mutation (at exon 16 splicing donor site). (B) Schematic representation of the pSLP3 minigene construct and putative splicing events. (C) PCR amplification of the (2) empty vector, the vector containing the minigene with the wt allele (3 and 4) and with the c.1428+1G>A mutation (5 and 6). Mk: 100pb molecular weight marker, (1) negative control.
